# The significance of programed cell death‐ligand 1 expression in vestibular schwannoma

**DOI:** 10.1002/brb3.3137

**Published:** 2023-06-27

**Authors:** Se A Lee, Susie Chin, Shin Jung, Kyung‐Hwa Lee, Kyung‐Sub Moon, Jong Dae Lee

**Affiliations:** ^1^ Department of Otorhinolaryngology‐Head and Neck Surgery Soonchunhyang University Bucheon Hospital, Soonchunhyang University College of Medicine Bucheon Republic of Korea; ^2^ Department of Pathology Soonchunhyang University Bucheon Hospital, Soonchunhyang University College of Medicine Bucheon Republic of Korea; ^3^ Department of Neurosurgery Chonnam National University Hwasun Hospital, Chonnam National University Medical School Hwasun Republic of Korea; ^4^ Department of Pathology Chonnam National University Hwasun Hospital, Chonnam National University Medical School Hwasun Republic of Korea

**Keywords:** immunochemistry, lymphocytes, neuroma acoustic, tumor‐infiltrating

## Abstract

**Background:**

The association between programed cell death‐ligand 1 (PD‐L1) and tumor‐infiltrating lymphocytes (TILs) in vestibular schwannoma (VS) has been investigated in a few studies. These published studies report a difference in the PD‐L1 positivity rate in malignant peripheral nerve sheath tumors. We examined PD‐L1 expression and lymphocyte infiltration in patients with VS who had undergone surgical resection and investigated the association between PD‐L1 expression and clinicopathological features.

**Methods:**

The expression of PD‐L1, CD8, and Ki‐67 in 40 VS tissue specimens was investigated using immunohistochemistry, and a clinical review of the patients was performed.

**Results:**

Of the 40 VS samples, 23 (57.5%) were positive for PD‐L1 and 22 (55%) were positive for CD8. No significant differences in age, tumor size, pure‐tone audiometry, speech discrimination, or Ki‐67 expression were observed between patients in the PD‐L1‐positive and PD‐L1‐negative groups. A higher level of CD8‐positive cell infiltration was observed in PD‐L1‐positive tumors than in PD‐L1‐negative tumors.

**Conclusion:**

We demonstrated that PD‐L1 was expressed in VS tissues. Although no correlation was identified between clinical characteristics and PD‐L1 expression, the association between PD‐L1 and CD8 was confirmed. Thus, additional research on targeting PD‐L1 is necessary to improve immunotherapy for VS in the future.

## Graphical abstract

The expression of PD‐L1 was associated with VS.

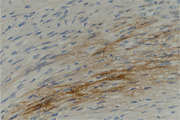

## 1 INTRODUCTION

Vestibular schwannoma (VS) is a benign tumor originating from the Schwann cells of the vestibular nerve (DeLong et al., 2011). In 95% of the patients with VS, the condition occurs unilaterally, although in neurofibromatosis type 2 (NF2), it may be bilateral (Møller et al., 2015). Also, in schwannomatosis, characterized by the development of multiple schwannomas, unilateral VS can be seen in patients with an LZTR1 mutation (Gonzalvo et al., 2011). Most patients with VS present hearing loss, tinnitus, or dizziness. A large tumor can cause headache, facial paralysis, and other cranial neuropathies, in addition to death from brainstem compression (Foley et al., 2017). Treatment of VS includes microsurgery, stereotactic radiosurgery, and conservative treatment through wait and scan. But there remains a need for effective pharmaceutical therapies. We have been conducting research on various mechanisms for the inhibition of VS tumor growth (Kim et al., 2016, 2015, 2022; Lee et al., 2012).

Programed cell death 1 (PD‐1) is a receptor expressed on the surface of T cells, which regulates the activation of T cells. This receptor interacts with PD‐1‐ligand 1 (PD‐L1). This binding suppresses T‐cell migration, proliferation, and secretion of cytotoxic mediators and restricts tumor cell killing (Akinleye & Rasool, 2019). Inhibitors of PD‐1 and PD‐L1 disrupt PD‐1/PD‐L1 axis, thereby reversing T‐cell suppression and enhancing endogenous antitumor immunity to unleash long‐term antitumor responses for patients with a wide range of cancers (Paterson et al., 2011). The expression of PD‐L1 in the tumor microenvironment is often associated with tumor‐infiltrating lymphocytes (TILs), among which CD8‐positive (CD8+) TILs may be the most potent (Zhou et al., 2018). Many clinical trials are being conducted to determine the therapeutic potential of PD‐L1 inhibitors in various tumors, either alone or in combination with other therapies (Balar & Weber, 2017; Wang et al., 2018).

Few studies have investigated the association between PD‐L1 and TILs in VS. The published studies are mostly related to NF2 or malignant peripheral nerve sheath tumors, and these studies report a difference in the PD‐L1‐positivity rate in these cancers (Perry et al., 2019; Shurell et al., 2016; Wang et al., 2018). We examined the expression of PD‐L1 protein and the infiltration of lymphocytes into the tumor tissue in VS patients who had undergone surgical resection and investigated the association between the expression of PD‐L1 and clinicopathological features.

## 2 MATERIALS AND METHODS

### 2.1 Patients and samples

Forty patients who underwent surgical resection for pathologically confirmed VS and for whom formalin‐fixed, paraffin‐embedded samples were available were included in this study. None of the patients received preoperative chemotherapy or radiotherapy. All clinical data were collected by retrospective review of medical charts and pathology records. The maximal linear diameter of the tumor was measured on a post‐gadolinium‐focused magnetic resonance image. Audiometric data were collected at the time of diagnosis with pure‐tone air‐conduction thresholds. Pure‐tone average (PTA) was calculated as outlined by the hearing committee of the American Academy of Otolaryngology—Head and Neck Surgery (AAO‐HNS) (American Academy of Otolaryngology, 1979), with average air‐conduction thresholds of 0.5, 1, 2, and 3 kHz. Hearing outcomes were categorized based on AAO‐HNS criteria (Committee on Hearing & Equilibrium, 1995), with serviceable hearing defined as Class A or B hearing with a PTA ≤50 dB hearing level (HL) and speech discrimination (SD) ≥50%. This study was approved by the Institutional Review Board at Chonnam National University Hospital (CNUHH‐2020‐252) and patient informed consent was waived.

### 2.2 Immunohistochemistry

Immunohistochemistry (IHC) was performed using whole‐tissue slides and antibodies against PD‐L1 (SP263; Ventana Medical System, Tucson, AZ, USA), CD8 (C8/144B, 1:200; Agilent Technologies, Santa Clara, CA, USA), and Ki‐67 (clone MIB‐1, 1:100; Dako, Carpinteria, CA, USA). IHC results were obtained using the OptiView Detection Kit (Ventana Medical System) and the UltraView Detection Kit (Ventana Medical System).

### 2.3 IHC analysis

IHC staining results were evaluated by one pathologist (S.C.) who was blinded to all clinical information. PD‐L1 staining was evaluated for partial or complete membrane expression in tumor cells, regardless of the staining intensity. The percentage of cells with membrane expression in the total number of tumor cells was calculated, and samples with >5% were considered positive (Bellmunt et al., 2015; Lipson et al., 2013; Xylinas et al., 2014). For CD8 staining, the number of intratumoral CD8+ lymphocytes was counted in 10 representative high‐power microscopic fields (magnification, 400×), then the percentage of CD8+ lymphocytes in the total intratumoral lymphocytes was calculated. The tumor background (nonspecific staining, e.g., in perivascular spaces), areas of necrosis, areas of hemorrhage, and artifactual staining were excluded from evaluation. CD8+ lymphocyte density was evaluated using a semiquantitative scoring system. Five categorical scales of the following levels based on the percentage of CD8+ lymphocytes were used: <1%, 1%‒5%, 5%‒10%, 10%‒20%, and >20%. Ki‐67 index was evaluated by calculating the percentage of positively stained nuclei per 100 tumor cells after counting at least 3000 tumor cells in each sample. Quantitative image analysis to evaluate Ki‐67 expression was performed by acquiring photomicrographs of five different microscopic fields (magnification, 200×), followed by evaluation of the acquired images with the image analysis software GenASIs HiPath (Applied Spectral Imaging, Carlsbad, CA, USA). High proliferation was defined as >2% Ki‐67 index.

### 2.4 Statistical analysis

All data were analyzed using SPSS software version 26.0 (SPSS Inc., Chicago, IL, USA). Chi‐square and Fisher's exact tests were used to identify a correlation between PD‐L1 expression and the clinicopathological characteristics among the groups. A *p*‐value of less than .05 was considered to be significant.

## 3 RESULTS

### 3.1 Patient characteristics

Forty patients with VS who underwent surgical resection were included in this study. The mean age of the patients with VS was 52.75 years. The patient clinical characteristics are shown in Table 1.

**TABLE 1:** Demographic and clinical characteristics of patients with vestibular schwannoma.

	Value (*n* = 40)
Mean age (years)	52.75 ± 13.46 (27−81)
Male:female	17:23
Right:left	21:19
Tumor size (mm)	31.73 ± 13.41 (9−63)
PTA (dB)	59.93 ± 37.19 (5−100)
SD (%)	43.88 ± 42.79 (0−100)

*Note*: Values are presented as mean ± standard deviation or number.

Abbreviations: PTA, pure‐tone average; SD, speech discrimination.

### 3.2 VS with PD‐L1‐positive and CD8‐positive TIL expression

**FIGURE 1:**
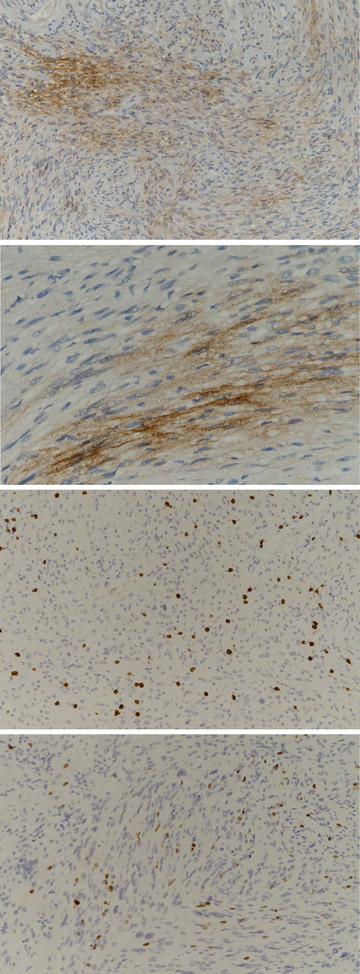
Representative images of immunohistochemical staining for programed cell death‐ligand 1 (PD‐L1) (SP263), CD8, and Ki‐67 in vestibular schwannoma. (A, B) Tumor cells exhibited membranous PD‐L1 expression with moderate to strong intensity (A: PD‐L1, ×200, B: PD‐L1, ×400). (C) CD8 staining revealed high density (>10%) of CD8 + lymphocytes (CD8, ×200). (D) Ki‐67 staining revealed high proliferative index, that is, high expression of Ki‐67 (×200).

IHC staining of the 40 VS specimens was used to investigate PD‐L1 expression on tumor cells (Figure 1A,B). Positive expression of PD‐L1 (>5% of tumor cells) was observed in 23 (57.5%) VS samples. We evaluated the density of cells with CD8+ expression in VS, and all specimens showed CD8 expression on the intratumoral lymphocytes (Table 2). There were significant differences in the number of PD‐L1‐positive tumors between low‐density (<10%) and high‐density (>10%) CD8+ tumor groups (*p* < .001) and this difference was maintained even when using a CD8+ positivity cutoff of 20% (*p* = .014). We defined high CD8+ lymphocyte density as >10% of the CD8+ lymphocytes (Figure 1C); 55% (22 samples) of the VS samples showed a high density of CD8+ lymphocytes.

**TABLE 2:** CD8 expression in vestibular schwannoma.

CD8 expression (%)	*n*
<1	0
>1, ≤5	12
>5, ≤10	6
>10, ≤20	15
>20	7

### 3.3 Association between the expression of PD‐L1 and clinicopathologic characteristics

Patient characteristics were correlated with PD‐L1‐positive and PD‐L1‐negative staining in VS specimens (Table 3). There were no significant differences in age, tumor size, PTA, or SD between the two groups. There also was no difference in the presence of serviceable hearing and PD‐L1 expression. We found a higher level of CD8+ cell infiltration in PD‐L1‐positive VS tumors than in PD‐L1‐negative tumors (*p* < .001). We also assessed the proliferation rate by Ki‐67 expression. High expression of Ki‐67 (>2%) was observed in 21 (52.5%) VS samples (Figure 1D), and there was no significant difference in Ki‐67 expression between the PD‐L1‐positive VS tumors and PD‐L1‐negative tumors.

**TABLE 3:** Summary of the association between PD‐L1 expression and patient characteristics.

	Negative PD‐L1 expression (*n* = 17)	Positive PD‐L1 expression (*n* = 23)	*p‐*value
Mean age (years)	53.76	52.00	.448
Tumor size (mm)	32.59	31.09	.808
PTA (dB)	55.53	63.17	.588
SD (%)	47.94	40.87	.808
Serviceable hearing			.523
Yes	10	10	
No	7	13	
CD8 + lymphocytes			<.001^*^
Low density (≤10%)	16	2	
High density (>10%)	1	21	
Ki‐67 index			.337
Low (≤2%)	10	9	
High (>2%)	7	14	

*Note*: Values are presented as mean or number.

Abbreviations: PD‐L1, programed cell death‐ligand 1; PTA, pure‐tone average; SD, speech discrimination.

## 4 DISCUSSION

The immune system provides a defense against antigens introduced from the outside and functions through an appropriate balance between activation and inhibition of the immune response (Naidoo et al., 2014). This immune response is responsible for carcinogenesis and antitumor activity. Immune checkpoints rely on cell surface molecules within the immune system that trigger coinhibitory stimuli of the immune response. Immune checkpoint therapy targets these immune checkpoints. The control and elimination of cancer by regulating the immune response through antibodies (Kyi & Postow, 2014) is a relatively new area of research. PD‐L1 and PD‐1 are among the most promising therapeutic approaches to various cancers.

It has been recognized that immune cells, such as macrophages and B and T lymphocytes, infiltrate the VS (Hannan et al., 2020). Evidence from both ex vivo and in vivo studies showed that inflammation and the immune microenvironment in VS play a key role in the pathophysiology of VS, and research on targeted molecular therapies for VS has drawn attention (Hannan et al., 2022; Lewis et al., 2019). Although VS is a benign tumor, it appears that the immune system checkpoint can be circumvented by expressing PD‐L1. In the present study, we investigated the association between VS and PD‐L1. Positive PD‐L1 expression was observed in 23 out of 40 (57.5%) of the VS samples, with a cutoff of 5% positive cells. There have been few studies on the association between PD‐L1 and VS. Inaguma et al. (2016) analyzed 5536 tumors, including germ cell, epithelial, mesenchymal, neuroectodermal, and lymphohematopoietic tumors. In mesenchymal tumors, PD‐L1 expression was the highest in peripheral schwannomas (89% of the samples), suggesting its role in the differential diagnosis of schwannoma. Archibald et al. (2010) reported that among 48 VS samples, 81% showed expression of B7‐H1 (B7 homolog 1, also known as PD‐L1). There are differences in the PD‐L1 positivity rates reported for VS. This is because the definition of the positivity rate of PD‐L1 expression is different in each study and the antibodies used in the studies are different.

**FIGURE 2:**
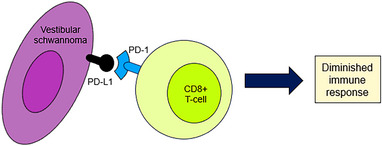
Schematic illustrating of PD‐1/PD‐L1 mechanism of action on vestibular schwannoma and CD8+ T cell.

PD‐L1 has been reported to act as a ligand that interacts with PD‐1 on activated T cells, thereby inducing apoptosis and inhibiting the proliferation and cytokine production of cells. Consequently, there is a diminished immune response to tumor cells and tumor growth can become unrestricted (Azuma et al., 2008; Hou et al., 2020). Many studies have reported that PD‐L1‐positive tumor cells are resistant to CD8+ cytolytic T‐cell‐mediated destruction (Azuma et al., 2008; Hirano et al., 2005). We found that 22 out of 40 (55%) of the VS samples showed high CD8+ lymphocyte density. VS samples with positive PD‐L1 expression showed a higher density of CD8+ lymphocytes than VS samples with negative PD‐L1 expression (Figure 2). As found in previous studies, PD‐L1 expression and CD8+ lymphocyte infiltration can be used as predictive biomarkers in response to treatment with immune checkpoint inhibitors.

We investigated the association between PD‐L1 in VS and the clinicopathological characteristics of the cells and found no significant correlation between PD‐L1 expression and age, tumor size, PTA, or SD. Archibald et al. (2010) reported that patients with poor hearing at the time of surgery tended to show higher levels of B7‐H1 than patients with better hearing, although no significant difference between the groups was found. Our study revealed similar results. There was no significant correlation between PD‐L1 and PTA or SD. Additionally, there was no difference between the presence of serviceable hearing and PD‐L1 expression.

We also assessed the association between PD‐L1 expression and tumor size, and there was no significant difference in PD‐L1 expression between the two groups. Archibald et al. (2010) compared the tumor size and B7‐H1 expression and found no significant correlation. Recently, it has been reported that VS tumor volume or progressiveness is associated with the concentration of PD‐L1 (Bi et al., 2020; Perry et al., 2019). Since our study investigated the correlation between the tumor size at the time of surgery and the expression of PD‐L1, additional studies on the characteristics associated with tumor progression are needed. We assessed the expression of Ki‐67 in VS, which is marker of high degree of tumor cell proliferation. Ki‐67 expression has been used to evaluate other tumors of the central nervous system. It has been reported that VS tumors with Ki‐67 labeling showed high proliferative activity, short doubling time, and high recurrence or regrowth after resection (Bedavanija et al., 2003; Prueter et al., 2019; Yokoyama et al., 1996). In the present study, the high expression of Ki‐67 was observed in 21 samples (52.5%) of VS. There was no significant association between Ki‐67 and PD‐L1 expression.

VS is a characteristic tumor of NF2. Although microsurgery and stereotactic radiation remain the mainstay of treatment for progressing VSs, observation is an acceptable option for newly diagnosed and nongrowing tumors. Despite the slow progression of the disease, no pharmacological treatments have been approved for VS or NF2, a fact that highlights the need to develop effective and well‐tolerated pharmaceutical therapies. Tumors with baseline infiltration of CD8+ cytotoxic lymphocytes are generally considered good candidates for checkpoint inhibitor therapy. Although there are still challenges in terms of side effects or other burdens such as high cost, we investigated the possibility of using PD‐L1 as a therapeutic agent for VS. Our study motivates future clinical studies of PD‐L1 for the treatment of VS and NF2.

The present study has a few limitations. This was a retrospective study with a small sample size, which may have led to bias in the data. In addition, PD‐L1 IHC was conducted using only one antibody. In measuring the size of VS, linear measurement rather than volumetric quantification was used, but the size measurement may not be accurate. Further experiments are needed for humanized mouse model for improved in vivo evaluation of several PD‐1/PD‐L1 checkpoint inhibitor drugs. These investigations would provide the possibility of potential therapeutic modalities.

In conclusions, we demonstrated that the expression of PD‐L1 protein was associated with VS. Although there was no correlation between clinical characteristics and PD‐L1 expression, the association between PD‐L1 expression and CD8+ lymphocyte density was confirmed. Additional research on targeting PD‐L1 in VS is needed in order to further develop immunotherapy for VS.

## CONFLICT OF INTEREST STATEMENT

The authors declare no conflicts of interest.

### PEER REVIEW

The peer review history for this article is available at https://publons.com/publon/10.1002/brb3.3137.

## Data Availability

Data sharing not applicable as no data generated.
